# Microglia retard dengue virus-induced acute viral encephalitis

**DOI:** 10.1038/srep27670

**Published:** 2016-06-09

**Authors:** Tsung-Ting Tsai, Chia-Ling Chen, Yee-Shin Lin, Chih-Peng Chang, Cheng-Chieh Tsai, Yi-Lin Cheng, Chao-Ching Huang, Chien-Jung Ho, Yi-Chao Lee, Liang-Tzung Lin, Ming-Kai Jhan, Chiou-Feng Lin

**Affiliations:** 1 Department of Microbiology and Immunology, School of Medicine, College of Medicine, Taipei Medical University Taipei 110, Taiwan; 2 Translational Research Center, Taipei Medical University, Taipei 110, Taiwan; 3 Department of Microbiology and Immunology, College of Medicine, National Cheng Kung University, Tainan 701, Taiwan; 4 Center of Infectious Diseases and Signaling Research, National Cheng Kung University, Tainan 701, Taiwan; 5 Institute of Basic Medical Science, College of Medicine, National Cheng Kung University, Tainan 701, Taiwan; 6 Department of Nursing, Chung Hwa University of Medical Technology, Tainan 717, Taiwan; 7 Department of Pediatric, College of Medicine, National Cheng Kung University, Tainan 701, Taiwan; 8 Department of Pediatric, School of Medicine, College of Medicine, Taipei Medical University, Taipei 110, Taiwan; 9 The Ph.D. Program for Neural Regenerative Medicine, College of Medical Science and Technology, Taipei Medical University, Taipei 110, Taiwan; 10 Graduate Institute of Medical Sciences, College of Medicine, Taipei Medical University, Taipei 110, Taiwan

## Abstract

Patients with dengue virus (DENV) infection may also present acute viral encephalitis through an unknown mechanism. Here, we report that encephalitic DENV-infected mice exhibited progressive hunchback posture, limbic seizures, limbic weakness, paralysis, and lethality 7 days post-infection. These symptoms were accompanied by CNS inflammation, neurotoxicity, and blood-brain barrier destruction. Microglial cells surrounding the blood vessels and injured hippocampus regions were activated by DENV infection. Pharmacologically depleting microglia unexpectedly increased viral replication, neuropathy, and mortality in DENV-infected mice. In microglia-depleted mice, the DENV infection-mediated expression of antiviral cytokines and the infiltration of CD8-positive cytotoxic T lymphocytes (CTLs) was abolished. DENV infection prompted the antigen-presenting cell-like differentiation of microglia, which in turn stimulated CTL proliferation and activation. These results suggest that microglial cells play a key role in facilitating antiviral immune responses against DENV infection and acute viral encephalitis.

Four serotypes of dengue virus (DENV), a mosquito-borne flavivirus, cause 390 million infections annually^1^. Approximately 3–14 days after infection, patients present symptoms including fever, severe headache, pain behind the eyes, muscle and joint pain, and rash. This stage of the illness is called dengue fever. Only 0.5–1% of patients progress to severe dengue hemorrhagic fever/dengue shock syndrome (DHF/DSS), which can be fatal. Patients who succumb to DHF/DSS frequently present early altered neurological consciousness, hypothermia, gastrointestinal bleeding, concurrent bacteremia, pulmonary edema, renal/hepatic failure, subarachnoid hemorrhage, and shock^2^, all of which show an association with diabetes, allergy, and hypertension^3^. The pathogenesis of DENV infection is determined by a number of virulence factors, including viral strain and viral load, cytokine storm, antibody-dependent enhancement, and immune evasion through escape from antiviral interferons and cytotoxic T lymphocytes (CTLs)^4^,^5^. The DENV RNA genome encodes a polyprotein precursor that is proteolytically cleaved into three structural proteins (capsid, premembrane, and envelope) and seven nonstructural proteins (NS1, NS2A, NS2B, NS3, NS4A, NS4B, and NS5) that control not only viral replication but also pathogenesis^5^. Furthermore, it is well known that humoral immunity is involved in severe dengue pathogenesis following a second infection. Antiviral agents and vaccine development should therefore be designed to target viral proteins^6^.

The WHO^7^ has designated new categories of dengue disease, including dengue with or without warning signs and severe dengue other than DHF/DSS, which includes central nerve system (CNS) and multiple organ involvement. The ability of DENV to invade the CNS by crossing the blood-brain barrier (BBB) passively or actively and its neuroinvasive capacity suggest that DENV can promote encephalitic neuropathogenesis; this has been confirmed by cerebrospinal fluid analysis of leukocyte counts and the detection of DENV antigen or RNA^8^,^9^,^10^. However, the neuropathogenesis of DENV infection requires further investigation with regards to the neurological manifestations experienced by severe dengue patients. These may be the result of direct neurotoxicity by DENV, hepatic failure, or the involvement of hemorrhagic complications, including thrombocytopenia, intracranial bleeding, prolonged shock, and hyponatremia. For dengue-associated neurological manifestations, including both encephalitis and encephalopathy, neuromuscular complications and neuro-ophthalmic involvement have been reported^11^. The clinical symptoms of patients with dengue encephalitis include diminished consciousness, headache, dizziness, disorientation, seizures, and behavioral symptoms^10^,^11^,^12^. However, a lack of *in vivo* immunocompetent disease models that mimic clinical findings retards the successful development of anti-dengue therapies.

Intracerebral inoculation with DENV in mice results in viral replication in the brain, leading to encephalitis, behavioral changes, and lethality^13^. A neuroadapted strain of DENV inoculated intraperitoneally in postnatal mice induces fatal encephalitis accompanied by limb paralysis and postural instability concomitant with efficient viral replication in the brain that likely occurs as a result of general and localized plasma leakage through the BBB^14^. In the brain, microglial cells, neurons, oligodendrocytes, and endothelial cells can be infected with DENV^14^,^15^; however, the effects of DENV on these cells remain undefined. Brain resident macrophage-like microglia cells are speculated to be targets of DENV infection that ultimately induce inflammatory activation *in vitro*^16^. However, the *in vivo* role of the microglia remains unclear. In general, microglial cells maintain immune homeostasis in the brain by triggering scavenging, phagocytosis, cytotoxicity, antigen presentation, synaptic stripping, promotion of repair, and extracellular signaling. Similar to peripheral macrophages, which have previously been shown to act as antiviral immune cells against DENV infection^17^, microglial cells are also critical for CNS inflammation as the first and foremost form of active immune defense during viral infection^18^,^19^. This study investigated the role of DENV-infected microglia *in vivo*, particularly with regards to CNS inflammation, neurotoxicity, encephalitis, and antiviral immune defense.

## Results

### Encephalitis-like DENV-infected mice exhibit local viral multiplication, neural impairment, CNS inflammation, hippocampal neurotoxicity, and BBB destruction

**Figure 1:**
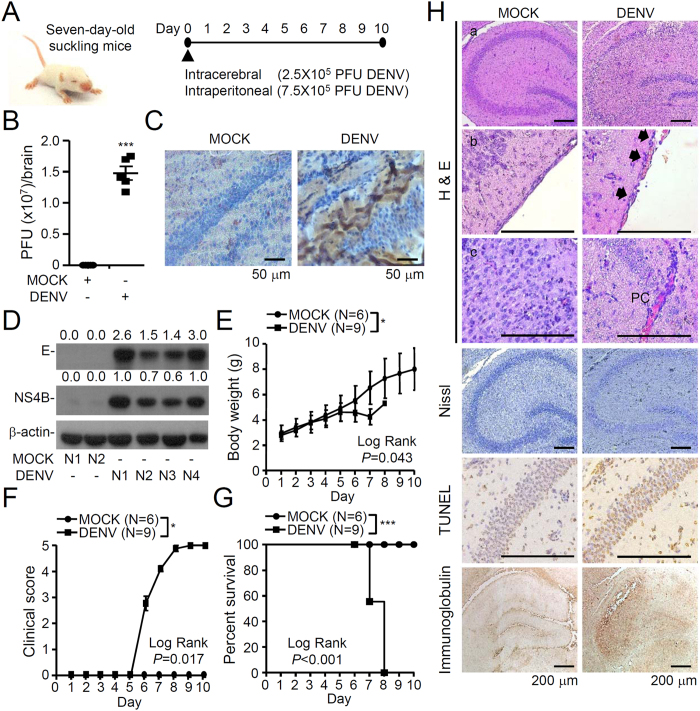
Viral replication status, clinical scores, survival rates, and neuropathological findings in DENV2-infected mice. (**A**) Seven-day-old ICR suckling mice were inoculated with DENV2 PL046 by concurrent intracranial and intraperitoneal injections. (**B**) Plaque assay for viral titers in the brain tissues of mice on day 7 post-infection (n = 5). Representative immunohistochemical staining of viral dsRNA (**C**) and Western blot analysis of viral proteins E and NS4B (**D**) confirming DENV infection. Time-kinetic changes in body weight (**E**), clinical score (**F**), and survival rate (**G**). *P < 0.05 and ***P < 0.001. (**H**) Histopathological changes were evaluated in the hippocampus and cerebrum. Neuropathies were characterized by a loss of hippocampus neurons, as shown by hematoxylin & eosin staining (H & E), Nissl staining, TUNEL assays, infiltration of immune cells (arrows), perivascular cuffing (PC), and immunoglobulin staining (open arrows) of hemorrhagic blood vessels.

Previous animal models of DENV infection used immunocompromised mice to facilitate DENV infection and replication^20^,^21^. To study the involvement of neuroinflammation and to verify the interplay between the virus and the host immune system, an immunocompetent mouse model of DENV infection was utilized and modified from the current models of DENV encephalitis-like infection^13^,^14^. To create an encephalitis-like mouse model, seven-day-old ICR suckling mice were inoculated concurrently with DENV serotype 2 (strain PL046) through intracerebral microinjection and intraperitoneal injection (Fig. 1A). We exploited a series of methods for detecting virus replication, including plaque assays (Fig. 1B), dsRNA immunostaining of viral RNA (Fig. 1C), and Western blot analysis of viral proteins E and NS4B (Fig. 1D). According to our results, DENV causes significant infection and replication in mouse brains 7 days post-infection. We next monitored a time-dependent change in body weight, clinical score, and the survival rate of the infected mice over 10 days post-infection. A significant decrease in body weight (Fig. 1E) and a significant increase in clinical score (Fig. 1F) occurred in the DENV-infected mice compared to the mock-infected mice 6 days post-infection. The survival rate in the DENV-infected mice was 55% on day 7 post-infection, and all of the mice died on day 8 post-infection (Fig. 1G). The data indicate an encephalitic model of DENV infection, leading to neural impairment following viral replication.

DENV-infected mice have been known to present with CNS inflammation accompanied by neurotoxicity^22^. In our study, an increase in paralysis and mortality was observed in DENV-infected mice, which provided insight into the neuropathological features within the infected mouse brains. Interestingly, DENV-infected mice showed intense neuronal loss from the CA1 to CA2 area of the hippocampus, as shown by H&E and Nissl staining (Fig. 1H). Further TUNEL staining confirmed neuronal cell apoptosis within the hippocampus. Multifocal areas of mononuclear cell infiltration and perivascular cuffing were observed in the DENV-infected mouse brains. During DENV infection, breakdown of the BBB has been reported^14^,^23^. In DENV-infected mice, immunoglobulin staining demonstrated an increase in BBB permeability. These findings show the involvement of CNS inflammation, neurotoxicity, and BBB destruction in DENV-induced encephalitis-like disease in mice.

### Microglia can be targeted and activated by DENV infection *in vivo* and *in vitro*

**Figure 2:**
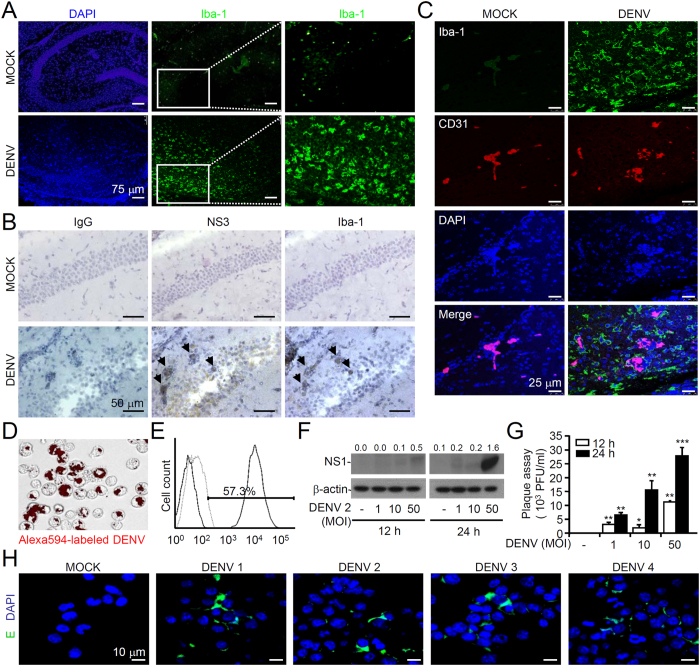
DENV infects and activates microglia *in vivo* and *in vitro*. (**A**) Representative confocal fluorescent immunostaining of Iba-1 (*green*), a marker for activated microglia, in DENV2 PL046-infected brains of ICR mice day 7 post-infection. DAPI is a nuclear stain (*blue*). (**B**) Representative AEC-based immunohistochemical staining of the viral NS3 protein and Iba-1 (*red*, arrowheads) in the serial sections of brain tissues. Hematoxylin is a nuclear counterstain (*blue*). (**C**) Representative confocal fluorescent immunostaining of Iba-1 (*green*) and CD31 (red) in DENV-infected brains. DAPI is a nuclear stain (*blue*). Murine microglial BV2 cells were inoculated with AlexaFluor 594-labeled DENV (MOI = 50) (*red*) for 2 h post-infection. A representative fluorescent image of viral entry is shown (**D**), and flow cytometric analysis was performed for quantification as a percentage (**E**). The MOIs of DENV infection were evaluated over a time course in BV2 cells by measuring viral NS1 protein expression (**F**) and by plaque assays (**G**). The values are the mean ± SD of three independent experiments. *P < 0.05, **P < 0.01, and ***P < 0.001, compared to mock. (**H**) Representative confocal fluorescent immunostaining of the viral E protein (*green*) in BV2 cells infected by the four serotypes of DENV. DAPI is a nuclear stain (*blue*).

Microglial cells are the resident macrophages in the brain and are likely infected by DENV because they possess the appropriate DENV receptors^24^. In DENV-infected mouse brains, fluorescent immunostaining of Iba-1, a marker of activated microglia, was utilized to show the large number of activated microglia, which are characterized by flat, ramified, and round amoeboid cells in the hippocampus; the presence of these cells leads to neuronal loss (Fig. 2A). As show by immunocytochemical staining of the viral NS3 protein, DENV may infect Iba-1-positive microglia in the injured hippocampal regions (Fig. 2B). We noted that NS3-positive cells are also present within the Iba-1 negative cell population, suggesting the ability of DENV to infect microglia *in vivo*. Furthermore, observations were made using a fluorescent confocal microscope that show activated microglial cells (Iba-1-positive) localized around and close to vessels (CD31-positive) (Fig. 2C). With regards to microglia being targets of DENV infection *in vitro*^16^, our work confirms DENV infection and viral entry, as shown by the uptake of Alexa Fluor 594-labeled DENV (Fig. 2D), flow cytometry (Fig. 2E), viral NS1 protein expression (Fig. 2F), and virus release (plaque assays) (Fig. 2G) in murine microglial cells (also known as BV2 cells). Additionally, all four serotypes of DENV caused infection in BV2 cells (Fig. 2H). These results indicate that DENV can infect microglial cells both *in vivo* and *in vitro*.

### Depletion of microglia enhances viral replication and mortality in DENV-infected mice

**Figure 3:**
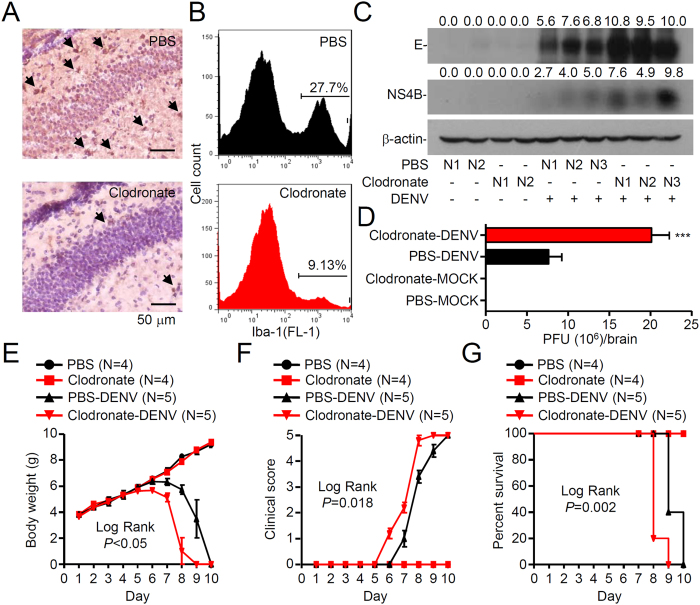
Depletion of microglia enhances viral replication and neuropathies, leading to death in DENV-infected mice. Liposome-encapsulated clodronate was used to deplete microglia in five-day-old ICR suckling mice for two days, followed by DENV infection. Liposome-encapsulated PBS was used as a control. Representative AEC-based immunohistochemical staining of Iba-1 (red, arrowheads) in brain sections is shown (**A**), and flow cytometric analysis of Iba-1 is expressed as a percentage in brain sections (**B**). Hematoxylin is a nuclear counterstain (*blue*). Western blot analysis of the viral E and NS4B proteins (**C**) and a plaque assay of viral replication (**D**) showing the effects of microglial depletion on DENV infection are shown. The values are the mean ± SD of three independent experiments. ***P < 0.001, compared to mock. Time course changes in body weight (**E**), clinical score (**F**), and survival rate (**G**) are shown.

The above findings and previous studies^15^,^16^ all suggest that microglial cells are targets of DENV infection, although a possible role for microglia-regulated CNS inflammation in DENV infection has been speculated. To determine if microglia are required for DENV-induced encephalitis, clodronate liposomes were used to deplete microglial cells^25^. Immunohistochemical staining of Iba-1 showed a decrease in microglial cells around the hippocampus after delivering clodronate liposomes (Fig. 3A). Following flow cytometric analysis, the number of Iba-1-positive microglia was further quantified (Fig. 3B). We next examined the role of microglia in DENV infection. Unexpectedly, depletion of microglia increased viral protein expression (Fig. 3C) and viral replication (3-fold increase in replication) (Fig. 3D). In DENV-infected mice depleted of microglial cells, we observed an early loss of body weight (Fig. 3E), increased clinical scores (Fig. 3F), and heightened mortality (Fig. 3G). These results suggest that activated microglia may confer antiviral responses and play a neuroprotective role against the progression of neurological impairment caused by DENV infection.

### Functional maturation and differentiation of microglia into APCs is associated with the production of antiviral CD8-positive CTL responses during DENV infection of the CNS

**Figure 4:**
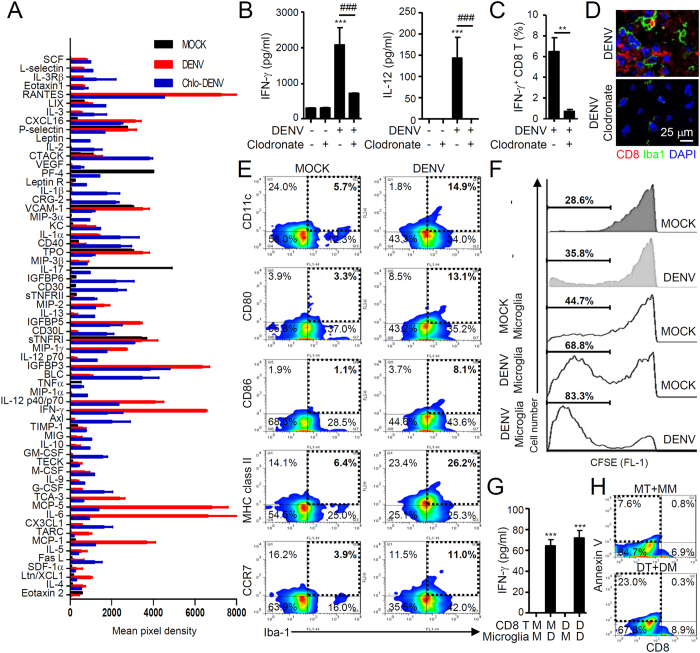
DENV infection induces the production of cytokines and chemokines and the differentiation of microglia into APCs, leading to antiviral T cell responses. Tissue supernatants and mononuclear cells infected with DENV with or without depletion of microglia were collected (n = 3). (**A**) A membrane antibody array containing 62 different cytokine antibodies was utilized to detect the secretion of soluble factors. The values are shown as the mean pixel density. (**B**) ELISA analysis was further carried out to detect IFN-γ and IL-12 production. ***P < 0.001 and ^###^*P* < 0.001. Flow cytometric analysis of IFN-γ-expressing CD8+ T cells (**C**) and representative confocal fluorescent immunostaining of CD8 (*red*) and Iba-1 (*green*) (**D**) show the activation and infiltration of CTLs, as well as their interaction with microglia. DAPI is a nuclear stain (*blue*). **P < 0.01. (**E**) Representative flow cytometric analysis of mononuclear cells, with or without DENV infection, was performed by staining for specific cell surface markers (Iba-1-FITC, CD11c-PE, CD80-PE, CD86-PE, MHC class II-PE, and CCR7-PE), and the results are shown as a percentage. CD8 T cells and Iba-1-positive microglia were purified by MACS from mock- and DENV-infected mice on day 7 post-infection (n = 5). CD8 T cell proliferation (**F**) and ELISA analysis of IFN-γ production (**G**) were measured following a mixed lymphocyte reaction performed using an *in vitro* inoculation of CFSE-labeled CD8 T cells and microglia with or without DENV infection, as indicated, for 72 h. ***P < 0.001. (H) Representative flow cytometric analysis of Annexin V staining shows the percentage of cell death in CD8^−^ microglia. M, Mock; MT, Mock CD8 T; MM, Mock microglia; D, DENV; DT, DENV-infected CD8 T; DM, DENV-infected microglia.

Microglia-derived CNS inflammation may contribute to neurotoxicity induced by viral infection^18^,^19^. However, our results showed that the presence of microglia slowed DENV infection, leading to neuropathies (Fig. 3). To address the question of whether DENV infection causes cytokine and chemokine production that results in microglia activation as part of the CNS immune defense system, we next investigated a specific role for microglia in confronting DENV infection. In contrast to the brains of mock-infected mice, the brains of DENV-infected mice with or without microglial cell depletion showed signs of cytokine and chemokine expression. The levels of several cytokines and chemokines, including RANTES, IL-12, IFN-γ, MCP-1, and MCP-5, were altered following DENV infection (Fig. 4A). ELISAs confirmed that depletion of microglia reduced the DENV-induced production of IFN-γ and IL-12 (Fig. 4B). Because most of the microglia-associated cytokines and chemokines may act as antiviral agents against DENV infection, we anticipated an antiviral response from the activated microglia. We found that depletion of microglia reduced the infiltration of IFN-γ-positive CD8 T cells (Fig. 4C). Additionally, DENV-induced CD8 T cell recruitment and interaction with activated microglia were halted by microglial cell depletion (Fig. 4D). These findings suggest an important role for activated microglia in antiviral immune responses during DENV infection of the CNS.

Infection of neuroadapted DENV can induce systemic immune responses^26^, while CD8-positive CTLs are required for cellular immune defense against systemic DENV infection^27^. Upon viral infection, activated microglial cells are able to process and present endogenous viral epitopes to T cells and competent APCs^28^. We therefore investigated whether DENV infection activates APC-like properties in microglia in response to antiviral CD8 T cells. To evaluate this possibility, we used flow cytometry to quantify the APC-related molecular expression patterns in microglia extracted from the brains of mock- or DENV-infected mice. The results revealed that the expression of CD11c, CD80, CD86, MHC class II, and CCR7 was increased 7 days post-infection (Fig. 4E). Furthermore, purification of microglia from DENV-infected mice in mixed lymphocyte reaction cultures resulted in the proliferation of CD8 T cells (Fig. 4F) and the production of IFN-γ (Fig. 4G). Cytotoxic CD8 T cells possess lytic effector molecules, such as granzymes and perforin, which are known to induce apoptosis^29^. DENV-infected microglia died in the presence of activated cytotoxic CD8 T cells (Fig. 4H). These findings demonstrate that DENV infection triggers the APC-like differentiation and maturation of activated microglia, which are essential for cytotoxic CD8 T cell proliferation and activation.

## Discussion

Although the neurological complications associated with severe dengue disease show CNS involvement and neurological dysfunction, the neuropathogenesis of dengue encephalitis is still unclear^11^. There are several emerging and unresolved questions still open for investigation, including the length of behavioral changes, the neuroinvasive capacity of DENV, the neurovirulence of different DENV serotypes, the routes of infection in the brain, the cells in the brain targeted by DENV and their possible roles in infection, immune defense in the brain, and the pathogenic role of CNS inflammation related to BBB destruction and intracerebral hemorrhaging. To answer these questions, several murine models of DENV infection in the brain have been created^13^,^14^,^26^. Together with our findings from this study, creating an animal model of DENV infection is important for the future study of DENV neuropathogenesis.

Overcoming the issues raised by immunodeficient mouse models is necessary to investigate the involvement of immune regulation in DENV-infected brains. Clinical observations and both *in vitro* and *in vivo* experimental studies have demonstrated that DENV efficiently infects CNS-associated cells. Several DENV receptors have been determined, including heparin sulfate, CD14, DC-SIGN, GRP78, HSPs, laminin receptor, heat shock proteins, beta3 integrin, mannose receptor, and C-type lectin domain family 5, member A (CLEC5A, MDL-1)^30^. After receptor binding, DENV infects target cells through receptor-mediated endocytosis^5^. Some DENV receptors, such as CD14, GRP78, and HSP70/90, are also expressed in microglial cells, as reported previously^24^. Similar to macrophages acting as the major DENV target cells in the periphery^31^, it is speculated that microglia are the major target cells of DENV in the brain. We created an immunocompetent mouse model in this study, and our data support the infection of microglia in the brain by DENV. This is also supported by other findings in a fatal dengue patient and in mice^14^,^15^. An infection model of Japanese encephalitis virus, a typical neurotropic virus, showed that microglial cells serve as a viral replication platform and lead to indirect neuronal killing via the secretion of TNF-α, resulting in neuronal cell death^32^. However, very little is known about the exact role of microglial cells in DENV-induced neurological complications. Although previous studies have hypothesized a pro-inflammatory and pathogenic role for activated microglia in viral neurotoxicity^16^,^19^, *in vivo* evidence is still needed. Our work shows, for the first time, an antiviral role for DENV-infected microglia in an encephalitis-like murine model. We were surprised to find that DENV-infected microglia acted as antiviral cells by taking on an APC-like phenotype to induce CD8-positive CTL responses. Consistent with our findings, a previous study showed that pharmacologically depleting peripheral macrophages increases DENV replication, suggesting an antiviral role for macrophages^17^. However, the roles of peripheral macrophages in controlling DENV infection are still controversial, particularly with respect to skin macrophages, which are the main local targets of DENV infection for further replication^33^.

The APC-like differentiation of microglia has also been observed in mice with other viral infections of the CNS, and these transformed microglia act as antiviral agents^28^. While we showed that pharmacologically depleting microglia enhanced DENV infection and replication, leading to neurotoxicity and lethality, it is postulated that DENV-infected microglia may be neuroprotective. After evaluating the production of the antiviral cytokines IFN-γ and IL-12, CD8-positive CTL responses, and the differentiation markers expressed by APC-like microglia, we found that DENV-infected microglia caused antiviral CD8-positive CTL responses after conversion to an APC-like phenotype. In another model of DENV-infected encephalitic mice, an increase in the infiltration of CD8-positive T cells accompanied by the presence of IFN-γ was reported; IFN-γ plays a crucial role in protection against DENV infection in mice and protects against initial systemic and subsequent CNS disease^34^. The actual role of CD8-positive CTL responses in the brain after DENV infection has been studied, although depleting CD8-positive cells retards DENV-induced encephalitic lethality^27^. DENV-infected peripheral and systemic dendritic cells trigger antigen presentation to activate CD8-positive CTL responses, which elicit antiviral effects against DENV infection^35^. However, dendritic cell maturation and activation following DENV infection triggers not only antiviral immune responses but also dengue pathogenesis^36^,^37^. The mechanism by which DENV induces dendritic cell or APC-like microglia maturation remains under investigation; however, dendritic cell differentiation signals cannot promote the APC-like differentiation of microglia but could promote anti-inflammatory properties in microglia^38^. Based on our findings, strengthening the activation of APC-like microglia may confer antiviral responses against both DENV infection and the progression of dengue encephalitis.

It is clear that DENV infection may lead to CNS manifestations, particularly an encephalitis-like illness, although its clinical course and prognosis are usually favorable^39^. Although the cases are limited, the possibility of an annual global DENV outbreak should be taken into consideration. In this work, as well as in other studies^13^,^14^, DENV-infected mice showed symptoms of reduced mobility, limbic seizure, limbic weakness, paralysis, and lethality 7 days post-infection. Markedly, DENV-infected mice also showed localized plasma leakage within the BBB. Because mortality is frequently high in animal models, we believe that the viral load and additional factors in humans may influence DENV-induced lethality. In acute dengue encephalitis, diminished consciousness, headache, dizziness, disorientation, seizures, and behavioral symptoms can be observed^10^,^11^. Our findings further uncover the involvement of the hippocampus in DENV infection, followed by the loss of hippocampal neurons, suggesting a long-term effect of DENV infection on learning and memory activity. It should be noted that neurons underwent apoptosis in DENV-infected brains, which contributed to anxiety-like behavior and hippocampal inflammation *in vivo*^22^. Indeed, some severe dengue patients exhibit symptoms of neurodegeneration, including amnesia, mania, depression, and seizures^40^,^41^. Our findings suggest that hippocampal neurons are also targets of DENV infection, indicating that DENV infection has a direct neurocytotoxic effect, as demonstrated previously^42^. The interplay between DENV infection and host factors, including effector cells and immune responses, needs further investigation.

## Methods

### Ethics statement

All animal studies were performed according to the rules of the Animal Protection Act of Taiwan, and all protocols were approved by the Laboratory Animal Care and Use Committee of National Cheng Kung University (IACUC #104062). The animals were raised and cared for according to the guidelines established by the Ministry of Science and Technology, Taiwan.

### Cells, Virus Strains, and Reagents

BV2 immortalized murine microglial cells, obtained from Dr. C. C. Huang (Department of Pediatrics, National Cheng Kung University, Tainan, Taiwan), were grown in Dulbecco’s Modified Eagle’s Minimal Essential Medium (DMEM; Invitrogen Life Technologies, Rockville, MD) supplemented with 10% heat-inactivated fetal bovine serum (FBS; Invitrogen Life Technologies), 50 U/ml penicillin, and 50 μg/ml streptomycin in a humidified atmosphere with 5% CO_2_ and 95% air. Baby hamster kidney (BHK) cells and *Aedes albopictus* C6/36 cells were cultured in DMEM containing FBS. C6/36 cell-adapted DENV serotypes (DENV1 8700828, DENV2 PL046 and 454009A, DENV3 8700829A, and DENV4 59201818) were obtained from the Centers for Disease Control in Taiwan and maintained accordingly^43^. Fluorescent DENV particles were prepared by labeling with AlexaFluor 594 succinimidyl ester (AF594SE, Molecular Probes, Invitrogen, Carlsbad, CA) according to a previous study^44^. The labeled viruses were purified using Amicon Ultra-15 PLTK Ultracel-PL Membrane (30 kDa) centrifugal filter units (Millipore, Billerica, MA) to remove excess dye. The entry of labeled DENV into cells was analyzed using a fluorescent microscope (BX51; Olympus, Tokyo, Japan) and quantified using a FACSCanto II Flow Cytometer (BD Biosciences, San Jose, CA).

### Infection models *in vivo* and *in vitro*

The *in vivo* infectious procedures were carried out according to a previous study with some modifications^45^. Seven-day-old ICR suckling mice, purchased from Charles River Laboratories (Wilmington, MA), were inoculated with DENV2 (PL0146) by intracerebral (2.5 × 10^5^ pfu) and intraperitoneal (7.5 × 10^5^ pfu) injections. To deplete microglial cells *in vivo*, liposome-encapsulated clodronate (FormuMax Scientific, Palo Alto, CA) was intracerebrally injected as previously described^25^. For *in vitro* infection, BV2 cells (5 × 10^5^ cells/ml) were re-suspended with DENV in the appropriate medium and incubated for 90 min at 37 °C. After 90 min, the cells were washed once with RPMI medium and re-suspended at a concentration of 5 × 10^5^ cells/ml for further incubation at 37 °C with 5% CO_2_.

### Encephalitic model assessment

Mice with neurological changes were evaluated as previously described to assess their scores, which were graded according to the severity of illness as follows: 0 for healthy; 1 for minor illness including weight loss, reduced mobility, and a hunchback body orientation; 2 for limbic seizure; 3 for moving with difficulty and anterior limb or posterior limb weakness; 4 for paralysis; and 5 for death^46^.

### Immunofluorescence and histological analysis

Mouse brains were prepared in tissue blocks and sliced. For histopathology, the tissue slices were fixed in 10% neutral-buffered formalin and embedded in paraffin wax. Sections (7 μm) or cells were stained with antibodies against dsRNA J2 (English and Scientific Consulting, Szirák, Hungary), DENV proteins E and NS3 (GeneTex, San Antonio, TX), CD8, IFN-γ, CD31, and activated microglial marker Iba-1 (Abcam, Cambridge, MA). DAPI was used for nuclear staining. Brain sections to be used for histology were stained with hematoxylin and eosin (H & E). Pathological changes, including encephalitis characterized by infiltration of immune cells, perivascular cuffing, hemorrhagic blood vessels, and vasculitis, were analyzed by pathologists. To detect brain lesions, a Nissl staining kit (MDS Analytical Technologies, CA) was used to analyze Nissl bodies in the cytoplasm of neurons after fixing and freezing in paraformaldehyde, as described previously^47^. Apoptotic cells were assessed by TUNEL (TdT-mediated dUTP Nick End Labeling) staining using an ApoAlert DNA fragmentation assay kit (Clontech, Mountain View, CA) according to the manufacturer’s instructions. IgG extravasation was detected by anti-mouse IgG conjugated with HRP (Chemicon International, Temecula, CA) as an indicator of BBB permeability, as described previously^48^.

### Plaque assay

Supernatants were collected from the brains of mock- or DENV-infected mice or DENV-infected BV2 cells. BHK-21 cells were plated onto 12-well plates (2 × 10^5^ cells/well) and cultured in DMEM for plaque assays, as described previously^43^.

### Western blot analysis

Total cell or tissue lysates were extracted and proteins were separated using SDS-polyacrylamide gel electrophoresis. The proteins were then transferred to a polyvinylidene difluoride membrane (Millipore). After blocking, the membranes were probed with the indicated antibodies (E, NS1, NS4B, and β-actin) and developed using an ECL Western blot detection kit (Pierce Chemical, Rockford, IL) according to the manufacturer’s instructions. The relative signal intensity was quantified using ImageJ software (version 1.41o) from W. Rasband (National Institutes of Health, Bethesda, MD) ( http://rsb.info.nih.gov/ij/).

### Isolation of primary mononuclear cells from the CNS and flow cytometric analysis

Mononuclear cells (MNCs) were isolated from the brains of mock- or DENV-infected mice as previously described^49^. Briefly, saline-perfused brains were homogenized using a 5 ml syringe with an 18–20 G needle in PBS and centrifuged at 200 × g for 15 min at 20 °C. Cell pellets were passed through a 70 μm nylon cell strainer, suspended in 37% (w/v) Percale (4 ml per brain), and then overlaid onto 70% (w/v) Percoll (4 ml) in 15 ml conical tubes; 30% (w/v) Percoll (4 ml) and HBSS (with phenol red, 2 ml) were added prior to centrifugation at 200 × g and 20 °C for 40 min without braking. After centrifugation, MNCs were harvested from the red fraction, which included the white section below the ring, and washed with PBS to remove residual Percoll. The isolated MNCs were resuspended in flow staining buffer (2% FBS and 0.1% NaN_3_ in PBS) containing human serum for FcR blocking and incubated for 15 min on ice. The MNCs were washed again with flow staining buffer and incubated with fluorochrome–conjugated anti-Iba-1, CD11c, CD80, CD86, MHC class II, CCR7, and CD8 mAbs. For intracellular cytokine staining, the cells were fixed with 4% (v/v) paraformaldehyde and permeabilized with 0.1% (w/v) saponin before incubation with IFN-γ mAbs. The cells were analyzed using a FACSCanto II Flow Cytometer. The data were analyzed using CellQuest Pro 4.0.2 software (BD Biosciences).

### Cytokine antibody array

Saline-perfused brains were homogenized in PBS using 5 ml syringes with 18–20 G needles and centrifuged at 200 × g for 15 min at 20 °C. Cell supernatants were collected and stored at −80 °C. The cytokine production profile was determined using a cytokine antibody array (Mouse Cytokine Antibody Array III M0308003; RayBiotech, Inc., Norcross, GA) and a Luminex Flowmetrix system according to the manufacturer’s instructions.

### ELISA

The concentrations of cytokines (IFN-γ and IL-12) in the lysates of brain tissues and cell-conditioned culture medium were determined using ELISA kits (R&D Systems, Minneapolis, MN) according to the manufacturer’s instructions.

### T cell stimulation assay

CD8 T cells were enriched to 90% purity from the spleens of mock- or DENV-infected mice by MACS (Miltenyi-Biotec, Auburn, CA) using anti-CD8a-conjugated microbeads. Purified CD8 T cells were washed with PBS and resuspended to 5 × 10^7^/ml in PBS containing 1 mM CFSE (Molecular Probes, Eugene, OR). The cell suspension was incubated at 37 °C for 10 min and immediately washed with cold RPMI 1640/10% FCS before plating, according to previous procedures^50^. Graded numbers of MNCs from mock- or DENV-infected mice were seeded in 96-well flat-bottom tissue culture plates and tested for their ability to re-stimulate T cells immediately after seeding. After 72 h of incubation, the CFSE-labeled CD8 T cells were collected and analyzed using a FACSCanto II Flow Cytometer.

### Statistical analysis

Statistical analyses for parametric data were performed using Student’s t-test (two groups) or one-way ANOVA (more than two groups) with Prism 6.0 (GraphPad). The data are presented as the mean ± standard deviation (SD). Significance was set at *P < 0.05,**P < 0.01, and ***P < 0.001.

## Additional Information

**How to cite this article**: Tsai, T.-T. *et al*. Microglia retard dengue virus-induced acute viral encephalitis. *Sci. Rep.* **6**, 27670; doi: 10.1038/srep27670 (2016).
